# The Auxiliary Role of Heparin in Bone Regeneration and its Application in Bone Substitute Materials

**DOI:** 10.3389/fbioe.2022.837172

**Published:** 2022-05-12

**Authors:** Jing Wang, Lan Xiao, Weiqun Wang, Dingmei Zhang, Yaping Ma, Yi Zhang, Xin Wang

**Affiliations:** ^1^ Department of Orthopaedic Surgery, Affiliated Hospital of Zunyi Medical University, Zunyi, China; ^2^ Centre for Biomedical Technologies, School of Mechanical, Medical and Process Engineering, Queensland University of Technology, Brisbane, Australia; ^3^ Australia−China Centre for Tissue Engineering and Regenerative Medicine, Brisbane, Australia; ^4^ Department of Hygiene Toxicology, School of Public Health, Zunyi Medical University, Zunyi, China

**Keywords:** heparin, nanomaterial, osteogenic, bone regeneration, bone morphogenic protein-2, bone morphogenic protein-4, heparan sulfate

## Abstract

Bone regeneration in large segmental defects depends on the action of osteoblasts and the ingrowth of new blood vessels. Therefore, it is important to promote the release of osteogenic/angiogenic growth factors. Since the discovery of heparin, its anticoagulant, anti-inflammatory, and anticancer functions have been extensively studied for over a century. Although the application of heparin is widely used in the orthopedic field, its auxiliary effect on bone regeneration is yet to be unveiled. Specifically, approximately one-third of the transforming growth factor (TGF) superfamily is bound to heparin and heparan sulfate, among which TGF-β1, TGF-β2, and bone morphogenetic protein (BMP) are the most common growth factors used. In addition, heparin can also improve the delivery and retention of BMP-2 *in vivo* promoting the healing of large bone defects at hyper physiological doses. In blood vessel formation, heparin still plays an integral part of fracture healing by cooperating with the platelet-derived growth factor (PDGF). Importantly, since heparin binds to growth factors and release components in nanomaterials, it can significantly facilitate the controlled release and retention of growth factors [such as fibroblast growth factor (FGF), BMP, and PDGF] *in vivo.* Consequently, the knowledge of scaffolds or delivery systems composed of heparin and different biomaterials (including organic, inorganic, metal, and natural polymers) is vital for material-guided bone regeneration research. This study systematically reviews the structural properties and auxiliary functions of heparin, with an emphasis on bone regeneration and its application in biomaterials under physiological conditions.

## 1 Introduction

Research into anticoagulant drugs has provided substantial evidence that heparin and its derivatives play an important role in the treatment of venous thromboembolism (VTE) (Piran and Schulman, 2016). Heparin was discovered in 1916, and from 1937 it was in use for the prevention of pulmonary embolism and later for the treatment of acute VTE. The binding of heparin to antithrombin Ⅲ enhances antithrombin III activity. This results in the formation of molecular complexes including thrombin and serine proteinases which inhibit the activity of coagulation factors Ⅱ and Ⅹ. Heparin exhibits rapid, long-lasting anticoagulation activity and low bioavailability, especially at low dosages (Zee et al., 2017). Low molecular weight heparin (LMWH) is derived from the depolymerization of heparin, and it was introduced as an anticoagulation drug approximately 10 years ago. Compared with unfractionated heparin (UFH), the advantages of using LMWH include a stronger anticoagulation effect, increased bioavailability, reduced risk of bleeding, and an enhanced safety profile. Moreover, the use of LMWH is known to reduce the risk of thrombocytopenia (HIT) compared with UFH, thereby allowing it to be used in pregnant women to treat VTE (Cosmi, 2016; Thaler et al., 2015). Furthermore, administering LMWH removes the need of coagulation tests as opposed to when UFH is used to treat VTE.

Long-term use of heparin has been linked to potential osteoporosis, which leads to poor bone healing (Schulman and Hellgren-Wångdahl, 2002; Alban, 2012). Miur et al. demonstrated that both UFH and LMWH reduced bone formation by decreasing osteoblasts and the osteoid surface, but only UFH increased bone resorption by increasing the osteoclast surface (Muir et al., 1996; Muir et al., 1997). The long-term use of UFH has been found to impede bone mineral density (BMD) resulting in increased risk of fracture, whereas LMWH treatment did not show any obvious effects on fracture risk (Schulman and Hellgren-Wångdahl, 2002; Pettilä et al., 1999; Monreal et al., 1994). Recent findings suggest that heparin can serve as an effective tool to modify bone substitute materials since the incorporation of heparin with biomaterials can improve bone tissue bioengineering. Current biomaterial delivery vehicles for carrying therapeutic growth factors (GFs) have limitations which lead to low affinity for GFs. Therefore, improved biomaterials capable of providing localized and persistent release of bioactive proteins are essential for bone regeneration (Hettiaratchi et al., 2014). Heparin is an attractive vehicle for GF delivery due to its ability to reversibly bind positively charged proteins required to maintain bioactivity and sustained release of GFs. However, the main challenge is how to ensure the grafted tissue substitutes with a sufficient blood supply. Interestingly, the application of heparin–chitosan coating can significantly enhance blood perfusion and re-endothelialization in the early stages of transplantation (Sun et al., 2011). Therefore, heparin is considered an important bioactive component in bone substitute material design. In the current review, we have summarized the structural properties and function of heparin and the role of heparin on bone regeneration, specifically its application in biomaterial development.

## 2 Heparin Structure

The molecular weight (MW) of heparin ranges between 3,000 and 30,000 Daltons. It is proposed that L-iduronic acid (IdoA), mainly IdoA2SO_3_, is the major aldehydic acid which heparin is composed of, while D-glucuronic acid (GlcA) and N-acetyl glucosamine (GlcNAc) are components of the heparin chain (Carlsson and Kjellén, 2012; Nahain et al., 2018). These studies, together with structural analyses of purified heparin, established the idea that heparin displayed structural microheterogeneity. Furthermore, studies have also described structural differences of heparin between species such as porcine and bovine (Bett et al., 2017). Also, the content and size of IdoA2SO_3_ and IdoA in heparin are different, which results in different lengths of the heparin chains (Crijns et al., 2021). Antithrombin (AT), a serine protease inhibitor, functions by binding to serine residues within serine proteases leading to their inactivation. AT-mediated inhibition was also found. Indeed, the ability of AT to inhibit serine protease could be significantly enhanced by heparin (Onishi et al., 2016). The anticoagulation activity of heparin is primarily based on its capacity to bind with AT, thereby increasing the AT-derived inhibitory effects on coagulants by several orders of magnitude. The inhibitory effects of heparin and AT vary and depends on the affinity of heparin with AT which can be variable. The antithrombin-binding region of heparin was identified as a pentasaccharide sequence, with the structure GlcNAc6S-GlcA-GlcNS3S6S-IdoA2S-GlcNS6S, with the 3-O-sulfate and 6-O-sulfate structures involved specifically in AT binding (Alekseeva et al., 2019). In summary, heparin exists as a linear, unbranched, and deeply sulfated GAG, which primarily consists of the helical structure (Mulloy et al., 1993).

## 3 Heparin in Biomaterial Design

### 3.1 Classes of Heparins

Heparin is mainly biosynthesized at the endoplasmic reticulum and Golgi apparatus of mast cells in the liver, intestine, and lungs. The anticoagulation activity of heparin is derived from its inhibitory effect on multiple proteins in the coagulation cascade. Heparin binds to AT and facilitates the inhibition of coagulation proteins including Factor Ⅱa and Xa. Upon heparin binding, AT undergoes a conformational change exposing a reactive loop that is subjected to factor Xa and thrombin to catalyze their inactivation. Heparin binds with AT and thrombin to form a ternary complex, which requires heparin to have at least 18 polysaccharide residues (Lane et al., 1984; Jonas and Quartermain, 2001).

Heparan sulfate (HS) is a linear polysaccharide composed of repeated disaccharide units, increased sulfation, and covalently linked to a core protein to form heparin sulfate proteoglycan. HS can bind to a variety of bioactive molecules, including GFs [such as heparin-binding epidermal growth factor (HB-EGF), fibroblast growth factor (FGF), and transforming growth factor-β (TGF-β)], chemokines, and morphogenetic proteins, which are linked to the core protein and function (Li and Kusche-Gullberg 2016). HS bound with GFs protects from protease degradation and promotes their ability to bind to their cognate receptors, thereby enhancing and prolonging the activity of GFs. HS has been used in fracture models to promote bone healing, suggesting it may be a useful tool in the treatment of non-union or delayed union fractures (Nozawa et al., 2018).

LMWH, which have an average MW ranging between 2 and 5 kDa have more desirable characteristics compared to UFH, including a longer plasma half-life, better bioavailability at low doses, and more predictable dose–response effects (Schwartz, 1990). The inhibitory effect of LMWH is mainly exerted *via* FXa and has little effect on thrombin in contrast to heparin. For this reason, LMWH can result in effective anticoagulation and reduced thrombocytopenia compared with heparin, which, due to its powerful thrombin-inhibitory effect, is the main cause of thrombocytopenia. This suggests that the use of LMWH has great value in clinical application (Gray et al., 2008). LMWH is expressed at lower levels in macrophages, endothelial cells, platelets, osteoblasts, platelet factor 4, and plasma proteins, which can reduce many problems generated by heparin, such as short plasma half-life, HIT, and osteoporosis (Mikhailov et al., 1999).

Ultralow molecular weight heparins (ULMWHs) consist of small heparin chains, many of which are homogeneous compounds, with a MW ranging between 1.5 and 3.5 kDa (Chandarajoti et al., 2016). ULMWHs are demonstrated to have increased bioavailability, longer plasma half-lives, reduced risk of bleeding, and osteoporosis (Hao et al., 2011) and were shown in one clinical study to have a greater safety margin reducing the risk of bleeding (Rico et al., 2011). ULMWHs are suggested to be more suitable than UFH for long-term clinical treatment and can even be administered subcutaneously to prevent or treat VTE (Alquwaizani et al., 2013).

### 3.2 Heparin Application in Nanomedicine

Heparins are heterogenous in sequence and consist predominantly of a disaccharide repeating structure. The IdoA residue is responsible for specific orientation of the *O-* and *S-* groups, which allows heparin to bind to a variety of important proteins, such as cytokines, growth factors, and morphogenetic proteins (Mulloy and Linhardt, 2001). The interaction of heparin with heparin-binding proteins depends on the ionic and hydrogen bonds between its sulfur group and the amino group of the protein. Protein bound with heparin can participate in biological processes to promote tissue growth and development (e.g., binding growth factors or morphogenetic proteins to stimulate bone regeneration) (Välimäki et al., 2021). The binding of heparin to proteins depends on the distance between basic amino acids as opposed to the configuration of the heparin-binding site. However, basic amino acids, arginine and lysine, were also found to be essential for hydrogen bonding. In addition, van der Waals forces and electrostatic interactions are required for the binding of heparin to proteins (Margalit et al., 1993).

Surface functionalization is very important for heparin-modified biomaterials. The ways in which heparin-functionalized surfaces can be formed includes 1) covalent binding of partial deamination of UFH molecules to the substrate of the amine-containing functional group by reductive amine (Biran and Pond, 2017); 2) the coating surface was treated with cationic polyamines, and then heparin was connected to the polyamines through ion interaction (Biran and Pond, 2017); 3) heparin binds to the material through covalent interaction and ion interaction (Diez-Escudero et al., 2018); 4) heparin and light reagent applied to the material under ultraviolet irradiation produced covalent-fixed heparin surface (Charron et al., 2022). The modification and pretreatment of heparin resulted in better performance, such as the reaction of heparin and 2-aminoethyl methacrylate to prepare heparin methacrylate containing two carboxyl groups; heparin cultivated with N-(3-dimethylaminopropyl)-N'-ethylcarbodiimide/N-hydroxysulfosuccinimide (EDC/ Sulfo-NHS) solution on ice (about 2–4°C) for 15 min can activate the heparin carboxylic group (Prokoph et al., 2012); low molecular weight heparin sodium solution was prepared by ionic water; heparin-AL (aldehyde-modified heparin) was obtained by reaction of NaIO_4_ with amino-glycerol–modified heparin (Roberts et al., 2016). The heparin aqueous solution was mixed and incubated with the material (e.g., chitosan) aqueous solution to the desired concentration, then the unbound heparin was eluted with PBS or balanced salt solution to obtain the preliminary heparin-modified materials. Heparin-bound growth factors (e.g., PDGF-BB, BMP-2, and TGF-β_1_) were immobilized to prevent their diffusion from the matrix, while heparin was immobilized on the surface of materials (Wang et al., 2015). Moreover, aqueous solution of materials (such as chitosan) were combined with aqueous heparin using magnetic stirring and ultrasonic treatment; free heparin and chitosan were removed by washing with distilled water; then the nanomaterials were obtained by supercentrifugation (14000rMP, 15min) (Tan et al., 2011).

The interaction between GFs [such as TGF-β, FGF-1, and bone morphogenetic protein 2 (BMP-2)] and heparin is mostly dependent on alkaline residues within GFs, ionic interaction of acid sulfate, and carboxylate groups of heparin (Hettiaratchi et al., 2020). As such, heparin and GFs can be modified and assembled into biomaterials to be delivered to the injured site for bone regeneration. The use of heparin (UFH or LMWH) in tissue engineering includes heparin-functionalized surfaces (*via* electrostatic interaction, physical encapsulation, or chemical fixation), nanohydrogels, polymer nanoparticles, heparin nanogel layers, and polyelectrolyte composite nanoparticles (Farokhzad and Langer, 2009). In recent years, heparin-modified nanomaterials have attracted more attention due to their improved stability, enhanced cell uptake capacity, and ability to facilitate targeted therapy. Heparin covers the surface of nanoparticles and sometimes becomes the structural unit of the materials (Gallagher et al., 2020). Heparin nanoparticles (synthesized by the emulsion method) can provide higher protein affinity and improved controlled release of GFs. Modification of heparin can greatly reduce the damage of secondary and tertiary protein structures (Liang and Kiick, 2014). In addition, composite nanoparticles can be prepared by mixing oppositely charged polymer solutions with polypositive/anionic solutions. These composite heparin-containing nanoparticles can be combined with high porosity nanostructures, such as chitosan, for binding, stabilization, and release of GFs (Laner-Plamberger et al., 2021).

**FIGURE 1:**
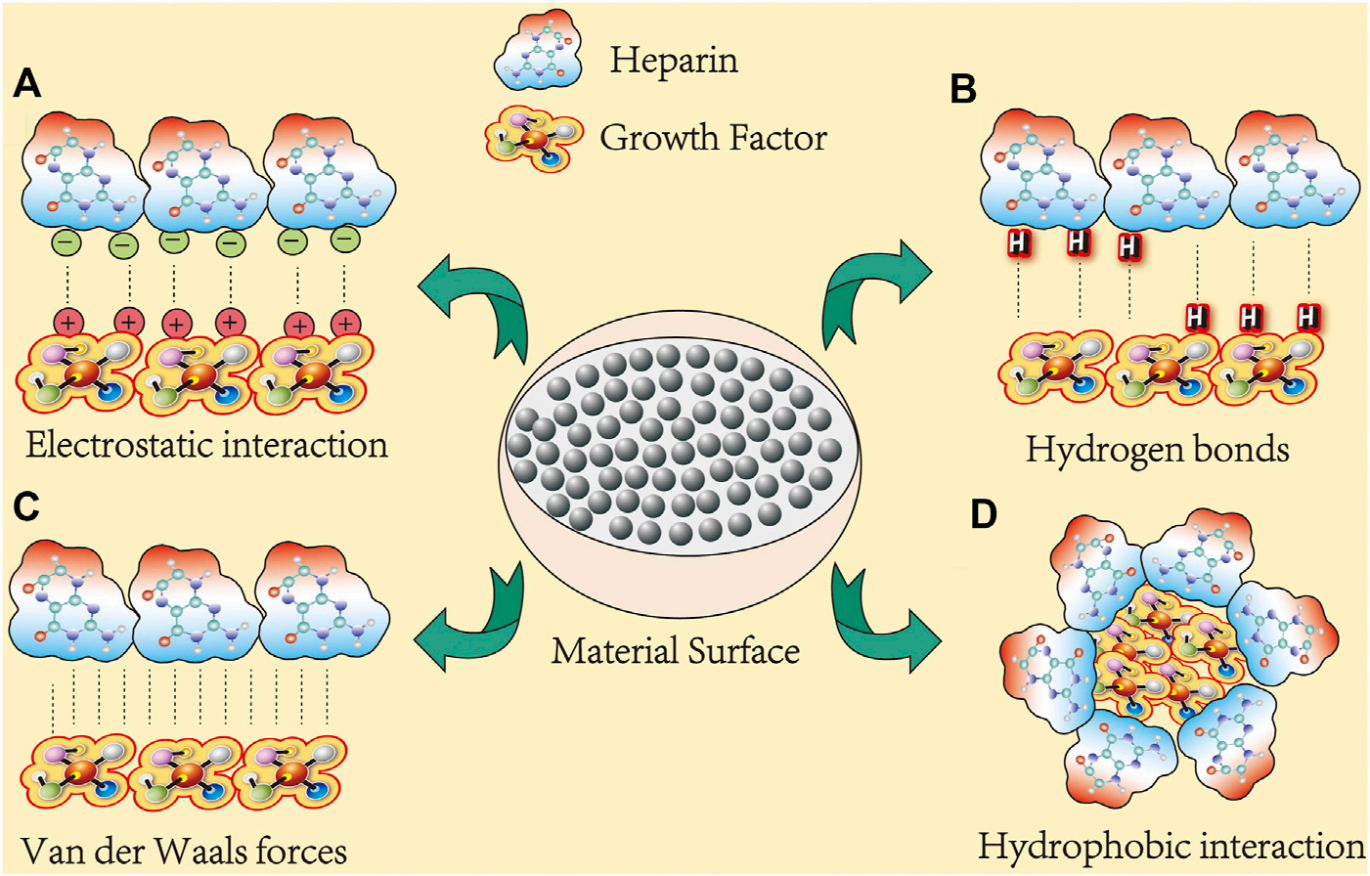
Heparin binds to growth factors in several ways. At least 300 membrane-associated proteins are known to bind to heparin (here, growth factors represent osteogenic proteins). Their interactions can be categorized into four classes: **(A)** electrostatic interactions: electrostatic interactions dominate most protein–heparin interactions. The negatively charged sulfate or hydroxyl groups in heparin are attracted to the positively charged lysine or arginine residues in the protein, thereby holding the molecules together. **(B)** Hydrogen bonds: polar residues, especially asparagine and glutamine, usually form hydrogen bonds with sulfate groups. For example, in FGF-1 and FGF-2, all three polar residues are involved in hydrogen bonding with sulfate groups, and the hydrogen bonding contributes most of the binding free energy. **(C)** Van der Waals forces: van der Waals force between heparin and protein molecules and include inducement force, orientation force, and dispersion force. **(D)** Hydrophobicity: the energy effect caused by the electrostatic forces and hydrogen bonds of polar groups in heparin and proteins cause polar groups to come together.

Currently, heparins used in bone tissue engineering mainly include UFH, LMWH, and HS. They exhibit similar biological properties, such as the storage and release of GFs although their chemical structures are different (Banik et al., 2021). Among them, LMWH is a popular choice in bone tissue engineering because of its excellent efficacy, reduced side effects, and optimized molecular size. For example, in a collagen sponge with LMWH-binding fibrin carrier system, LMWH polyelectrolyte carrier was designed for a more controlled release of slower growth factor (Facchetti et al., 2021). Figure 1 depicting several forces at which heparin interacts with growth factors.

## 4 Bioactive Functions of Heparin and its Effects on Bone Remodeling

### 4.1 Anticoagulation Activity and Other Effects of Heparin

As an effective anticoagulant, heparin has been used clinically for over 80 years, and it is still the primary medicine for prevention and treatment of VTE (Aláez-Versón et al., 2017; Torri and Naggi, 2016). With deep vein thrombosis and pulmonary embolism, the choice of heparin dosage depends on disease progression, heparin administered, and pre-existing medical problems. The subcutaneous dose is approximately 170–200 IU/kg for LMWH and 230–300 IU/kg for UFH (Konstantinides, 2014). These can be administered as a single dose or divided into two smaller doses delivered twice a day. After initial treatment, patients will be transitioned to vitamin K agonists (VKAs) or new oral anticoagulant treatments. The treatment is usually continued for 3 months or longer to guarantee a reduced risk of deep vein thrombosis (DVT) (Wells et al., 2014). LMWH is the treatment of choice for pregnant patients as it requires only twice-daily injections. VKAs are prohibited during pregnancy because of their teratogenic effects resulting in fetal abnormalities in the first trimester and risk of intracranial bleeding in fetuses in the third trimester with the capacity to cross the blood–brain barrier (Konstantinides 2014; Wells et al., 2014). Similarly, twice-daily administration of LMWH stabilizes plasma levels. Compared with patients who experience unprovoked VTE, patients with cancer are four times more likely to develop VTE. Therefore, the initial treatment of LMWH or VKAs should be extended to 3–6 months. Patients with non-progressed cancer can continue treatment indefinitely (Du et al., 2019). Heparin also reduced the clotting response and may reduce the risk of stroke by treating or preventing atrial fibrillation-related thromboembolic complications (Ezekowitz et al., 2018).

The non-anticoagulant properties of heparin are mainly reflected in its anticancer, anti-inflammation, and antivirus activities. Heparin and its derivatives inhibit the progression of cancer by generating antimetastatic effects as well as alleviating the hypercoagulable condition in cancer patients (Klerk et al., 2005). Heparin binds to P-selectin glycoprotein ligand-1 (PSGL-1) and blocks platelets to bind to cancer cells *via* mucin ligands on the cell surface of cancer cells, thus blocking their subsequent interaction with endothelium and reducing metastasis and tumor colonization (Mousa and Mohamed, 2004; Wahrenbrock et al., 2003; Borsig et al., 2001). Another common route through which cancer metastases occurs is *via* the lymphatic system. Recently, researchers have found a heparin derivative—a conjugation of LMWH and four bis-deoxycholates, which can reduce lymphangiogenesis by inhibiting vascular endothelial growth factor C (VEGF-C)–mediated phosphorylation of vascular endothelial growth factor receptor 3 (VEGFR-3) (Choi J. U et al., 2017). LMWH has been shown to improve 3- and 6-month survival in cancer patients (Icli et al., 2007; Prandoni et al., 1992; Gould et al., 1999), and heparin-like glycosaminoglycans (HLGAGs) have been reported to have similar effects (Kakkar et al., 2004). Several publications have demonstrated non-anticoagulation effects of heparin, such as binding to inflammatory factors, neutralization of the complement factor C5a, prevention of neutrophil chemotaxis and migration of leukocytes, and isolation of acute inflammatory proteins. Heparin can interact with many proteins involved in the pro-inflammatory and pro-coagulant cascade to guard against sepsis-associated inflammation and coagulation abnormalities (Li and Ma, 2017; Gockel et al., 2022; Lippi et al., 2022). In addition, in animal models of chronic myocarditis, heparin was found to be associated with low rates of myocardial inflammation and collagen deposition (Frizelle et al., 1992). The polyanion properties of heparin allow its binding to a variety of proteins, thus becoming an efficient inhibitor of viral attachment and plays a unique role in certain infectious diseases (including herpes simplex virus, Zika virus, and SARS-associated Vero cells) (Pouyan et al., 2021; Ghezzi et al., 2017). Other effects of heparin are summarized in Table 1.

**TABLE 1:** Biological roles of heparin.

Biological roles	Heparin-binding protein/Effect of heparin	References
Anticoagulation	Factors IIa, IXa, and Xa	Rosenberg (1975)
	Antithrombin (AT)	Rosenberg (1975)
Anti-Inflammation	Platelet growth factor 4, PGF4	Rabenstein (2002)
	Platelet factor 4	Arepally (2017)
	Interleukin 8	Rabenstein (2002)
	P-selectin, L-selectin	Nelson et al. (1993)
	MAC1	Peter et al. (1999)
Pathogen infection	HIV-1 envelope protein	Kamhi et al. (2013), Rabenstein (2002)
	Herpes simplex virus (HSV) envelope proteins	Rabenstein (2002), Kamhi et al. (2013)
	Hepatitis virus (B, C, and E)	Rabenstein (2002), Kamhi et al. (2013)
Growth factor binding	Transforming growth factors (TGFs)	Font Tellado et al. (2018)
	Platelet-derived growth factors (PDGFs)	Ishihara et al. (2019)
	Fibroblast growth factors (FGFs)	Spivak-Kroizman et al. (1994)
	Endothelial growth factors (EGFs)	Yu et al. (2013)
Angiogenesis	Vascular epidermal growth factor A (VEGF-A)	Ishihara et al. (2019)
	Angiopoietins/angiogenin	Ishihara et al. (2019)
	Platelet-derived growth factors (PDGFs)	Ishihara et al. (2019)
	Heparin-binding growth factor (HB-EGF)	Dong et al. (2020)
	Angiomodulin (AGM/TAF/mac25)	Kishibe et al. (2000)
Anticancer	Heparin inhibits angiogenesis and tumors	Cassinelli and Naggi (2016)
	Interaction between heparin and p-selectin may inhibit cancer metastasis	
Alzheimer’s disease	Sulfated GAG from the hippocampus of Alzheimer’s disease enhances growth factor and tau protein binding	Zhang W et al. (2019)
Acute coronary syndrome	Heparin in clinical prevention of acute coronary syndrome	Cassinelli and Naggi (2016)
Asthma	Anti-inflammatory, antioxidant, and mucolysis effects of heparin, when administered by inhalation, have the potential to alter disease progression in chronic obstructive pulmonary disease (COPD) and asthma	Shute et al. (2018)

### 4.2 Effect of Heparin on Bone Remodeling

Bone is a dynamic tissue which constantly undergoes a life-long remodeling process and comprises osteoblast-driven bone formation and osteoclast-driven bone resorption. Long-term use of heparin can lead to many adverse effects such as hemorrhage, HIT, general hypersensitivity reactions, elevated transaminase levels, and osteoporosis (Hemker, 2016; Arepally and Cines, 2020; Signorelli et al., 2019; Ludwig et al., 2006). Here, we focus on the effects of heparin on bone metabolism. Current studies demonstrate controversial results, wherein heparin was found to inhibit bone formation and contributed to the development of osteoporosis (Signorelli et al., 2019), while in another study, it induced osteogenesis by binding to BMP-2 and PDGF (Smith et al., 2018; Rindone et al., 2019). This part of the review will center on the effects of heparin on bone remodeling.

#### 4.2.1 Effects of Heparin on Bone Mineral Density

Long-term use of heparin has been shown to result in increased risk of osteoporosis and fracture due to decreasing BMD. Clinical studies demonstrated long-term use of UFH (> for 6 months) resulted in a significant decrease in BMD. A BMD analysis of 123 pregnant women with antiphospholipid syndrome treated with either UFH (*n* = 46) or LMWH (*n* = 77) revealed a 4% reduction in lumbar BMD during pregnancy. In addition, in a small case-control study, measurements of proximal femoral density decreased by ≥ 10% in 5 out of 14 pregnant women (35.7%). No one in the control group exhibited a decrease in BMD (*p* = 0.04), with the difference still statistically significant 6 months after delivery (*p* = 0.03) (Barbour et al., 1994). Similarly, Douketis et al. and Dahlman et al. found that long-term heparin therapy reduced BMD by 7 and 5%, respectively (Douketis et al., 1996; Dahlman et al., 1994).

The effect of LMWH on BMD is controversial. Some researchers found that using dalteparin did not correlate with reduced BMD during pregnancy (Rodger et al., 2007), and there was no discernible difference between LMWH-related bone loss and loss attributed to the physiological process of pregnancy (Carlin et al., 2004). Casele et al., also reported that the rate of bone loss in pregnant women receiving thromboprophylaxis was between 2 and 5%, and the results were similar for those administered with LMWH or UFH (H. Casele et al., 2006). However, a more significant loss in BMD was observed in patients receiving enoxaparin for a year or longer (Nelson-Piercy et al., 1997; Casele and Laifer, 2000; Greer and Nelson-Piercy, 2005). In addition, LMWH administration for at least 3 months also associated with bone loss and fractures (Carlin et al., 2004; Pettilä et al., 2002).

The mechanism underlying heparin-facilitated osteoporosis is only partially understood. Studies demonstrate that UFH and LMWH can both reduce osteoblast or osteoid formation (alkaline phosphatase is decreased), but only UFH increased osteoclast formation resulting in bone resorption (Muir et al., 1996). This can be largely attributed to the differences in the MW of heparin. Heparin fragments with an average MW < 3000 Daltons minimally affect osteoblast differentiation or bone mineralization. In addition, heparin can bind to osteoprotegerin (OPG) preventing its interaction with receptor activator of nuclear factor-κB ligand (RANKL), which impedes the OPG-RANKL axis while facilitating RANKL–RANK interactions to enhance osteoclastogenesis. Moreover, UFH with a high MW is more likely to form an obstacle in the space and has a higher affinity with OPG than LMWH (Li et al., 2016).

#### 4.2.2 Effect of Heparin on Bone Regeneration

##### 4.2.2.1 Transforming Growth Factor Family

Thus far, approximately a third of the TGF-β superfamily members including TGF-β1, TGF-β2, BMP-2, growth and differentiation factors (GDFs), and glial cell-line–derived neurotropic factor (GDNF) along with its two close homologues have been found to bind to heparin and HS (Rider and Mulloy, 2017). Most TGF-β family members show enhanced capacity to promote bone formation. Thus, heparin or HS indirectly facilitates osteogenesis by binding with the members of the TGF-β family.

###### 4.2.2.1.1 TGF-β1

TGF-β1, a universal growth factor, is involved in proliferation, migration, and differentiation. In bone, TGF-β1 signaling is a crucial key factor in cartilage and bone formation, remodeling, and maintenance. TGF-β1 promotes proliferation and chemotactic attraction of osteoblasts, enhances initial stages of osteogenic differentiation, facilitates production of extracellular matrix (ECM) proteins, stimulates type II collagen expression, stimulates chondrocyte precursors proteoglycan synthesis, reduces osteoblast apoptosis, and inhibits hematopoietic precursors (Janssens et al., 2005; Xu et al., 2020). TGF-β1 induces transcription factor Runx2 (a major driver of bone formation) in combination with BMPs during early stages of osteogenic differentiation (Liu et al., 2019). On the other hand, TGF-β1 also participates in regulating the recruitment of osteoclast precursors into the bone environment, which regulates differentiation and maturation of osteoclasts, bone resorption, and osteoclast apoptosis (Lee et al., 2019). Indeed, TGF-β1 enhances the osteogenic effect by promoting osteoblasts and inhibiting osteoclasts, but this effect can be influenced by many factors. Specifically, *in vivo*, the skeletal environment and the presence of other cytokines can alter the effects of TGF-β1 signaling (Bendinelli et al., 2019; Xie et al., 2020).

Heparin and highly sulfated HS can bind tightly to TGF-β1 enhancing the activity of TGF-β1. Unlike conventional cytokine–receptor interactions, this enhancement is due to the antagonistic action of α2-macroglobulin binding and inactivation of TGF-β1 (Gigi et al., 2012). In a study investigating fibrin extradomain-A (ED-A)–containing fibronectin (ED-A FN) and TGF-β–binding protein 1 (LTBP-1), Klingberg et al. proposed that the ED-A domain within ED-A FN promoted direct binding of heparin with LTBP-1. Furthermore, potential TGF-β1 binding was enhanced by heparin-mediated protein interactions within the FNIII12-13-14 (HepII) region in fibronectin. TGF-β1 activation was significantly reduced when the function of the ED-A domain was blocked with antibodies and competitive peptides (Klingberg et al., 2018). Together, this suggests that the heparin-enhanced TGF-β1 activity can be induced by specific domains in fibronectin. The structure and properties of heparin are crucial for TGF-β1 activity for many physiological processes. A competitive analysis of optical biosensors (Biacore) suggests that N- and 6-O-sulfate groups may play central roles in binding TGF-β1, whereas the 2-O-sulfate groups play a minor or a negligible role (Lee J et al., 2015). Thus, the effect of heparin on the TGF-β1 activity depends on the sulfated form of the polysaccharide. Furthermore, during osteogenic differentiation of human mesenchymal stem cells (hMSCs), cells treated with 1 ng/ml TGF-β1 and 10 μg/ml heparin expressed higher levels of TGF-β1 downstream effectors, phospho-SMAD2, and phospho-SMAD3 compared with TGF-β1 alone However, higher doses of heparin (40 μg/ml) or TGF-β1 (5 ng/ml) did not enhance phosphorylation of SMAD2 and SMAD3. This suggests that the role of heparin is to prolong, instead of enhance, TGF-β1–induced SMAD signaling. This supports the opinion that heparin protects TGF-β1 from inactivation by binding to α2-macroglobulin (McCaffrey et al., 1989; Lee J et al., 2015). In addition, heparin significantly stimulated TGF-β1 production at concentrations ranging from 50 to 400 ug/mL during valvular interstitial cell (VIC) differentiation in the presence of heparin-modified substrates resulting in changes in cell morphology as well as promoting tissue growth by increasing the absorption of serum proteins, especially TGF-β1 (Cushing et al., 2005).

###### 4.2.2.1.2 BMP-2

Since the discovery of BMP-2 as an effective osteoinductive cytokine in 1988, the BMP superfamily is well studied in vertebrate bone biology (Salazar et al., 2016). In bone tissue engineering, BMP-2 plays a critical role due to its strong osteoinduction capability (Hashimoto et al., 2020) and has been observed to interact with heparin. Glycoaminoglycans (GAGs) enhance the BMP activity and BMP-2 and has been shown to be more selective for heparin than similar GAG polysaccharides. The size of heparin and degree of sulfation is crucial for the binding and activity of BMP-2. BMP-2 can bind with heparin hexasaccharides (dp6) and octasaccharides (dp8), but decasaccharides (dp10) have been found to be the shortest chain length to activate effective heparin-dependent cellular reactions (Smith et al., 2018). In addition, the effect of 6-O-sulfation on BMP-2 binding and activity was very important, whereas the effect of 2-O-sulfation was not clear. The activity of BMP-2 in the presence of higher levels of HS was at least four times higher than when less HS was present (Tellier et al., 2015). In addition, induction of BMP-2 activity is mainly due to the stimulation of 6-O-sulfated chitosan (6SCS), and 2-N-sulfate was the lower activated subgroup. A low dose of 2-N, 6-O-sulfated chitosan (26SCS) significantly enhanced the alkaline phosphatase (ALP; an osteogenic protein) activity and BMP-2–induced mineralization as well has increased mRNA expression of ALP and osteocalcin (OCN). Increased chain-length and enhanced sulfation of 26SCS resulted in an increased ALP activity. Moreover, in the presence of 26SCS, Noggin (a BMP-2 antagonist) did not inhibit the BMP-2 activity. 26SCS showed a stronger synchronous effect on the biological activity of BMP-2 at low dosage. Compared with BMP-2 alone, the combination with 26SCS contributed to a larger amount of ectopic bone formation. Together, this suggests that 26SCS is a stronger stimulator of BMP-2 activity resulting in osteoblast differentiation (Zhou et al., 2009).

Hyper physiological doses of BMP-2 have been used clinically for bone regeneration in non-union fractures, large bone defects, and spondylodesis. Its application has been limited as it degrades quickly and causes side effects such as abnormal bone formation (heterotopic ossification). In combination with heparin, the dosage used can be reduced but increases the osteoinductivity of BMP-2 (Terauchi et al., 2019). The use of heparin microparticles (HMP) has been demonstrated to improve BMP-2 retention *in vivo* and improve protein transfer to bone defects due to its strong affinity with BMP-2. Consequently, heterotopic ossification was decreased and there was improved spatial localization of bone formation in large bone defects (Hettiaratchi et al., 2020). *In vitro* experiments demonstrated that heparin-dependent osteoblast differentiation was stimulated by homodimers (BMP-2 or BMP-4) and heterodimers (BMP-2/6 or BMP-2/7). This was suggested to be due to heparin continually supplying ligands to signal receptors expressed on cell membranes, thereby enhancing activity of BMP homodimers and heterodimers (Takada et al., 2003). In an *in vivo* study, Zhao et al. treated C2C12 myoblast cells with heparin to investigate the molecular mechanisms underlying the stimulation of the BMP activity. Heparin treatment augmented gene expression of BMP-2 and phosphorylation of SMAD1/5/8 at 24 h. Furthermore, degradation of BMP-2 was inhibited, half-life of BMP-2 was increased 20-fold, and Noggin failed to inhibit BMP-2 from binding to heparin. The combination of BMP-2 and heparin enhanced mineralized bone tissue compared with using BMP-2 alone. Therefore, heparin prevents degradation of BMPs and augments their osteogenesis effect *in vitro* and *in vivo* (Zhao et al., 2006). The negative regulator of BMPs, Noggin, is able to bind heparin sulfate proteoglycan (HSPGs) on the surface of cells resulting in localization at the plasma membrane where it retains its antagonistic functions and can bind to BMP-4. As such, the interaction between Noggin and HSPGs regulates the formation of the BMP activity gradient *in vivo* (Paine-Saunders et al., 2002).

MSCs are pluripotent stromal stem cells that play a significant role in bone healing. Heparin has been found to enhance WNT and FGF signal transduction in human embryonic stem cells (hESCs) to upregulate cell proliferation (Furue et al., 2008; Sasaki et al., 2008). Heparin increases WNT-induced signalling in osteoblast differentiation (Ling et al., 2010) and affects the differentiation of bone precursors as well as having a crucial role in stereotyping and osteogenic/adipogenic differentiation of primary human bone marrow stromal cells (hBMSCs). Simann et al. demonstrated that heparin treatment significantly increase mRNA expression and activity of ALP as well as enhancing mineralization and augmented levels of BMP-4 mRNA. In addition, heparin treatment partially inhibited adipogenesis differentiation and transformation of MSCs by decreasing the expression of adipogenesis markers and reducing the formation of lipid droplets. The authors showed heparin-mediated osteogenesis signal transduction not only on BMP pathways but also through reducing mRNA levels of the WNT pathway inhibitor, dickkopf1 (DKK1), and sclerostin (SOST), hence indirectly promoting bone formation (Simann et al., 2015). Therefore, heparin not only promotes osteogenic differentiation *in vitro* but also inhibits adipogenic differentiation and transformation. Different doses of heparin have distinctive effects on proliferation and pluripotency of hMSCs. Treatment of hMSCs with low doses of heparin (<200 ng/ml) exhibited pleiotropic effects on proliferation and signalling growth and differentiation pathways (including TGFβ/BMP, FGFs, and WNT). However, at high doses of heparin (≥100 μg/ ml), cell growth was inhibited, cell size increased (including nuclear area), and hMSCs became more senescent (Ling et al., 2016).

Various types of heparin analogues have been shown to participate in bone regeneration. A heparin-like synthetic polymer derived from dextran called RGTA was able to enhance the bioactivity of the heparin-binding growth factor (HBGF) *in vitro* and interact with HBGF released at the wound site and stimulate bone healing. RGTA increased the activity of ALP and parathyroid hormone reactive adenylate cyclase in MC3T3 pre-osteoblasts. RGTA was also able to enhance the ALP activity stimulated by BMP-2 and increases the response to parathyroid hormone stimulated by BMP-2 (Blanquaert et al., 1999). Furthermore, RGTA alone or in combination with HBGF stimulated the expression of osteoblast phenotypic characteristics. When HS was cocultured in the osteogenic medium, HS core protein gene expression, in particular glypican-3, was increased (Haupt et al., 2009). Heparin-induced bone formation is therefore dependent on specific HS chains, especially those containing glypican-3. Cell surface HSPGs are involved in BMP-induced osteogenesis by regulating the BMP activity and gradient formation. Studies have shown that HSPGs directly control the BMP-2–mediated transdifferentiation of C2C12 myoblasts into osteoblasts and regulate the osteogenic activity of BMP-2 by sequestering BMP-2 and mediate its internalization (Jiao et al., 2007). It was suggested by Fisher et al., that exogenous HS significantly increased the ability of BMP-2 to activate chondrogenesis and chondrogenic gene expression and decreased concentration of BMP-2 required to activate chondrogenesis. In addition, HS stimulated BMP-2–mediated SMAD1/5/8 phosphorylation, suggesting that HS increases the interaction of BMP-2 with its receptors. Heparinase treatment to degrade endogenous HSPG enhanced the chondrogenic capacity of BMP-2. Together, these results suggest that exogenous HS or heparinase can augment the chondrogenic capacity of BMP-2 by interfering with the interaction between BMP-2 and endogenous HSPG (Fisher et al., 2006).

Different sources of HS have pleotropic effects. HS and heparin are derived from the bone marrow stromal cell line; HS-5 increase BMP-2–stimulated osteogenesis in C2C12 myoblasts by increasing ALP activation and OCN mRNA expression, respectively. Furthermore, HS significantly enhanced BMP-2–stimulated bone formation *in vitro* and *in vivo* by elongating the half-life of BMP-2, decreasing the antagonism of Noggin, and regulating the distribution of BMP-2 on the cell surface. This suggests that bone marrow–derived HS is highly effective in bone formation and is better suited for bone regeneration by improving the delivery and bioavailability of BMP-2 (Bramono et al., 2012).

###### 4.2.2.1.3 BMP-4

Bone morphogenetic protein 4 (BMP-4) is known to be involved in the process of bone formation both *in vitro* and *in vivo* (Sun et al., 2021; Zhang et al., 2020). Interaction of heparin to the heparin-binding domain (HBD) has been demonstrated to enhance the osteogenic function of BMP-4, and the synthetic peptide sequence homologous to the residues 15–24 of HBD within BMP-4 can bind to heparin to exert osteogenic properties. hMSCs treated with the HBD peptide showed increased ALP expression and calcium phosphate crystal formation similar to the osteogenic effect of BMP-4. Similarly, the HBD peptide could increase the expression of osteoblast-specific genes, including ALP, osteopontin (OPN), and OCN, and induce osteoblast differentiation *via* the phosphorylation of ERK1/2 in a concentration-dependent manner. Treatment with heparinase blocked HBD peptide-induced osteogenic differentiation and inhibited phosphorylation of ERK1/2. This revealed that the HBD peptide may stimulate osteoblastic differentiation by binding to cell surface heparin and activating ERK1/2 signaling. In summary, the osteogenic efficacy of the HBD sequence of BMP-4 is similar to BMP-4 (Choi et al., 2010).

Chondroitin sulfate (CS) is a family of sulfated GAGs of which chondroitin sulfate E (CS-E) plays and important role in regulating the differentiation and mineralization of osteoblasts by binding to BMP-4. Compared with heparin, CS-E enhanced ALP activity and mineralization as well as cell growth and collagen deposition. The administration of exogenous (soluble) BMP-4 further enhanced the mineralization ability of CS-E (Miyazaki et al., 2008). Thus, signaling and activation of BMP-4 are regulated by both exogenous and endogenous GAG, which was depended on sulfate residues of GAG (Khan et al., 2008). The extracellular level of BMP is regulated by endocytosis, which reduces the amount of BMP-4 and reduces the osteogenic role of BMP-4. HSPGs on the cell surface are the main receptor for the internalization of BMP-4. Treatment with heparinase (to reduce HSPG synthesis) or supplementation with heparin (to inhibit BMP-4 binding to HSPG) can reduce BMP-4 internalization and increase BMP-4 concentration. Heparin thus promotes bone formation by increasing extracellular BMP-4 concentration *in vivo* (Kim C. L et al., 2018).

###### 4.2.2.1.4 BMP-7

Bone morphogenetic protein 7 (BMP-7), also termed osteogenic protein (OP-1), is a member of the TGF-β superfamily. In the clinic, BMP-7 is used as a GF to accelerate bone healing, and clinical trials suggest that BMP-7 treatment may be an alternative to autologous bone graft, the gold standard for treating non-union fractures (Kim Y et al., 2018; Sun et al., 2020). BMP-7 is also a HBGF that exerts unique osteogenic effects in the presence of heparin. HS and heparin chains bind specifically to BMP-7. Researchers used heparinase to neutralize HS on the cell surface to downregulate BMP-7–mediated phosphorylation of SMAD in osteoblasts. Chlorate inhibition of HS sulfation also resulted in disruption of SMAD phosphorylation. Thus, the combination of BMP-7 and HS on the cell surface is required for BMP-7 signaling (Irie et al., 2003). Similarly, heparin and HS also enhance BMP bioactivity. It was found that the activity of BMP-2/4/6/7 was enhanced by heparin, of which activity of BMP-2/6 and BMP-2/7 heterodimers increased more significantly. Complex formation produced by sulfated polysaccharides with BMP was mediated *via* negatively charged polysaccharide chains or basic amino acid chains of BMP in the culture medium and continued to provide ligands for its signal receptors (Takada et al., 2003).

Heparin and BMP-7 also stimulate the development of embryonic cartilage. Macias et al., used heparin as a carrier to implant BMP-2 and BMP-7 in chicken embryos and found that BMP-7 was strongly expressed in the perichondrium of developing cartilage, while BMP-2 mainly acted on the joint space. In addition, they may have a direct role in limb morphology, such as regulating the number and distribution of undifferentiated prechondrogenic mesenchyme and controlling the initial position of long bone cartilage (Macias et al., 1997). BMP-7 is expressed in sclerotome, hypertrophied chondrocytes, osteoblasts, and periosteum in human embryos. In addition to bone formation, it also maintains a high affinity for basement membrane components and plays other important regulatory roles in the embryo (Vukicevic et al., 1994).

**FIGURE 2:**
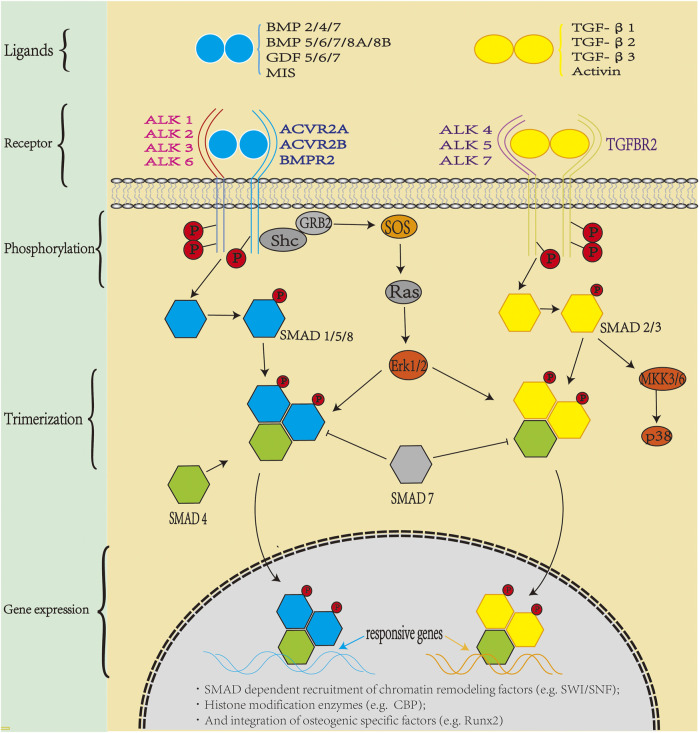
Bone morphogenic protein (BMP) signaling *in vivo*: TGF-β superfamily members include activin, inhibin, Mullerian inhibitor substance, and bone morphogenetic proteins (BMPs). BMP is the largest subfamily, with more than 30 BMP ligands in humans. BMPs transduce signals through a polymeric cell surface complex of which there are two classes, type I and type Ⅱ receptors. Both type I and type II receptors are single-channel transmembrane proteins with an intracellular serine/threonine kinase domain. Type II receptors phosphorylate type I receptors after ligand assembly, and the activated type I receptor recruit specific SMAD1/5/8 (blue pathway) and SMAD2/3 (yellow pathway), which combine with SMAD4 to form trimers which translocate to the nucleus. SMADs bind to DNA and recruit chromatin remodeling factors and tissue-specific transcription factors to regulate gene expression.

Different binding sites of heparin and BMP-7 can regulate the retention time of BMP-7, and an increased retention time has shown advantages in clinical application. The N-terminus of BMP-7 was replaced by the heparin-binding area of BMP-2, which increased the binding ability of the new protein to heparin. Moreover, *in vitro*, 100 ng/ml of BMP-7 increased the binding ability of heparin by approximately 20% compared with the untreated group (Nematollahi et al., 2013). Soluble BMP-7 protein was transferred into the periplasmic space of *Escherichia coli*, resulting in a monomer of approximately 16 kDa, which at a concentration of 500 ng/ml, binds to 50% more heparin than wild type. Therefore, BMP-7 with abundant binding sites may be more effective in osteogenesis (Nematollahi et al., 2012). The *in vivo* signal transduction process of BMP is illustrated in Figure 2:

##### 4.2.3.2 PDGF-BB

Blood vessels are essential for the development, growth, and remodeling of bone tissue. Anatomically, blood vessels provide bone tissue with nutrients, GFs, and other substances that enhance bone development and reconstruction. Blood vessels also transport the metabolites of bone tissue and ensure the stability of the bone microenvironment (Peng Y et al., 2020). Platelet-derived growth factor-BB (PDGF-BB) has been suggested to be beneficial for bone formation since it is demonstrated to stimulate angiogenesis during bone regeneration (Xie H et al., 2014) and local treatment of PDGF-BB increased angiogenesis and stimulated bone healing (Gao et al., 2021).

PDGF-BB contains a C-terminal heparin-binding sequence which binds heparin and has been shown to be important for the recruitment of pericytes during vascular development, since deletion of the heparin-binding sequence inhibited PDGF-BB retention and pericyte recruitment *in vivo*. The degree of sulfation is an inhibiting factor for the activity of PDGF-BB. Decreased N- sulfation reduced PDGF-BB binding *in vitro* and resulted in pericyte detachment or delayed pericyte migration as well as diminish PDGF-BB signaling directed cell migration. Therefore, the recruitment of pericytes requires an appropriate N-sulfate domain to retain PDGF-BB and stimulate PDGF-BB signaling (Abramsson et al., 2007). PGDF functions by activating a specific tyrosine kinase of the PDGF-α/β receptor. Low concentrations of heparin enhanced PDGF-BB–stimulated PDGF-α receptor phosphorylation in a dose-dependent manner. In CHO667 cells, heparin disaccharide treatment induced maximum (6-fold) phosphorylation of the PDGF-α receptor. Heparin enhanced PDGF-BB–induced mitogen-activated protein kinase (MAPK) and Akt activation and increased the chemotaxis of CHO667 cells to PDGF-BB. Therefore, heparin regulates PDGF-α phosphorylation and downstream signal transduction induced by PDGF-BB (Rolny et al., 2002).

Scaffolds for bone regeneration need to induce the inward growth of blood vessels. Porous scaffolds with a covalently bound heparin coating were demonstrated to be effective for *in vivo* delivery of PDGF-BB and VEGF. Both VEGF and heparin increased vascular ingrowth at 10 days; however, after 2 months, PDGF-BB–mediated delivery (but not VEGF) resulted in a significant increase in vascularization compared with heparin alone. Therefore, the use of a porous scaffold covalently combined to heparin resulted in a differential release of VEGF and PDGF-BB resulting in rapid and sustained vascular regeneration in the scaffold (Davies et al., 2012). In another study, heparin-bound fibrin was found to continuously release GFs that had a high affinity to heparin (Yang et al., 2011). Furthermore, heparin could potentially be used as a carrier for PDGF-BB continuous release in bone defects. PDGF-BB release was found to be prolonged by heparin-conjugated poly(lactic-co-glycolic acid) nanospheres, resulting in accelerated angiogenesis at the wound site (La and Yang, 2015).

**FIGURE 3:**
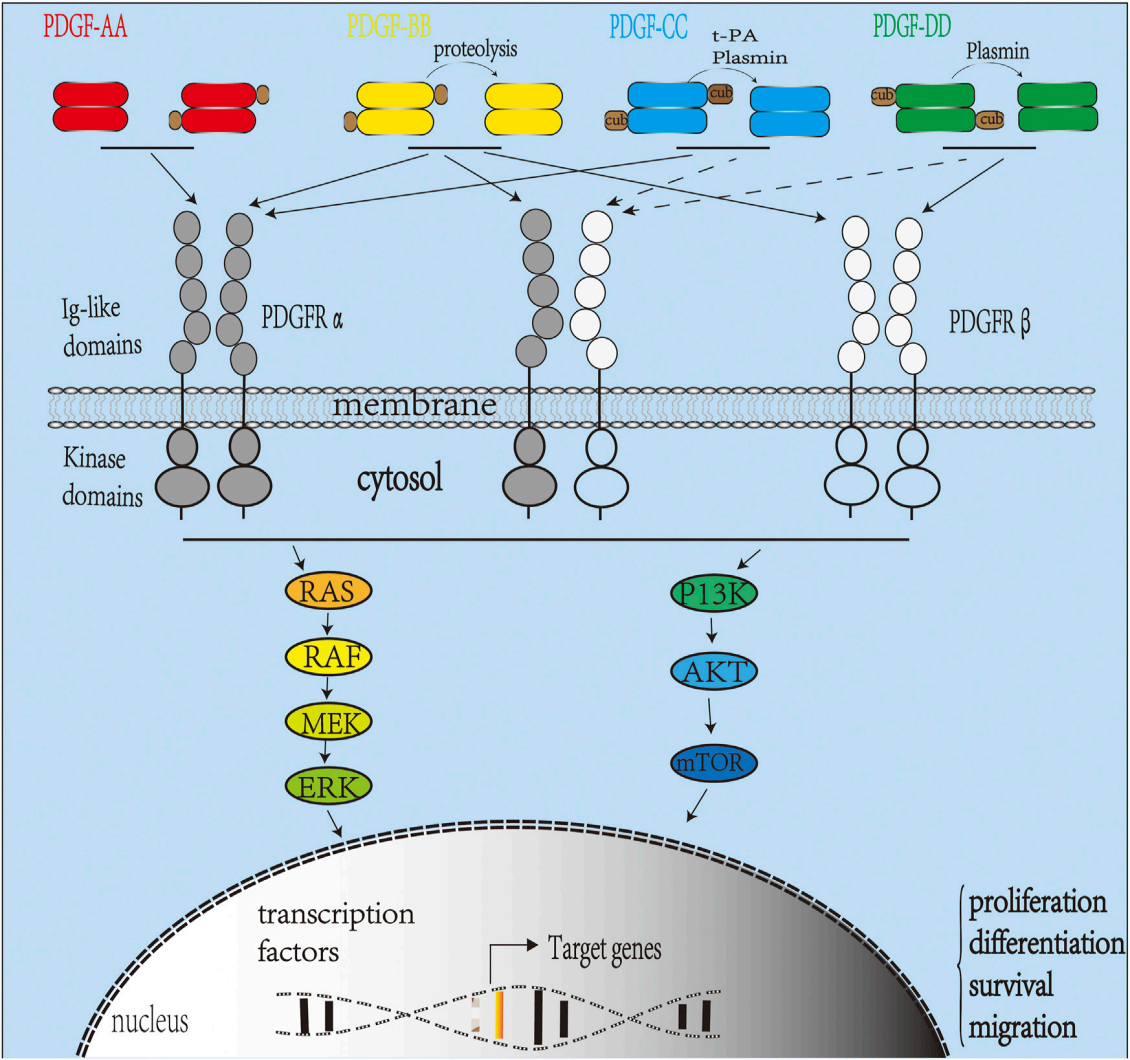
Platelet-derived growth factor (PDGF) signaling pathway *in vivo*. The PDGFR family includes PDGFRα and PDGFRβ. Activation of the receptor complex is associated with ligand binding leading to dimerization of PDGFRα and PDGFRβ forming homodimers or heterodimers. Ligands PDGF-C and D are secreted as inactive homodimers and require cleavage by tPA (PDGF-C), uPA (PDGF-D), or matriptase to be activated. PDGF-AA binds and activates only PDGFR-αα homodimers, while PDGF-BB can bind and activate PDGFR homodimers or heterodimers. PDGF-CC binds and activates PDGFR-αα and PDGFR-αβ. PDGF-DD activates PDGFR-ββ and, in some cases, PDGFR-αβ (Demoulin and Essaghir, 2014). Upon activation, intracellular tyrosine kinase domains of PDGFR autophosphorylate activating intracellular signaling pathways such as the RAS-MAPK and P13K pathways.

In another study, recombinant human platelet-derived growth factor (rhPDGF-BB) was fixed on the surface of heparinized titanium (Hep-Ti). When the rhPDGF-BB complex was combined with heparin, ALP activation and OCN mRNA levels increased. Moreover, it showed anti-inflammatory properties demonstrated by the downregulation of TNF-α and IL-6 at the transcript level (Huh et al., 2011). In addition, studies have shown that physiological concentrations of PDGF-BB can directly enhance the osteogenic effect of adipose stem cells (ASCs). Heparin-conjugated decellularized bone matrix promoted binding to PDGF-BB and after 3 months enhanced bone formation when grafts were implanted into critical-size skull defects in rats. Therefore, heparin-bound decellularized bone matrix can promote osteogenic signal transduction from PDGF-BB to ASC and stimulate ASC-mediated bone regeneration (Rindone et al., 2019). The *in vivo* signal pathway transduction of PDGF is illustrated in Figure 3.

##### 4.2.3.3 FGF-2

Fibroblast growth factor 2 (FGF-2), a classic member of FGF-1 subfamily, is expressed from a variety of cells and regulates proliferation, differentiation, migration, and cell survival. FGF-2 is also a major player in bone development, bone formation, and fracture repair (Yamaguchi and Rossant, 1995). Not only does it act as a strong mesoderm inducer during embryogenesis, but its receptor is also strongly expressed in developing bones (Kimelman and Kirschner, 1987). It is also expressed continuously in osteoblasts and stored in the ECM (Hurley et al., 1994).

FGF-2 binding and activation of its cognate receptor tyrosine kinases (FGFR-1/2/3/4) results in pleotropic effects: binding of FGF-2 to FGFR *via* the HS glycosaminoglycan binding site stimulates proliferation of BMSCs *via* the ERK1/2 pathway (Choi et al., 2008); FGF-2 signaling promotes Runx2 activity through FGF-2–mediated activation of the MAPK pathway (Long 2011). FGF-2 induces osteoblast and chondrocyte differentiation through the ERK1/2 pathway (Lai et al., 2001). The bioactivity of FGF-2 can be controlled through its binding to HS to promote the formation of ligand–receptor complexes. FGF-2–interacting HS variant (HS2), isolated from embryos, increased the proliferation and capacity of hMSC (Dombrowski et al., 2009). In addition, HS8 obtained from HS affinity isolation from porcine mucous membranes has higher FGF-2 binding affinity, upregulating FGF signaling, and hMSC proliferation *via* FGFR-1 (Wijesinghe et al., 2017). Therefore, affinity-purified HS variants have better FGF-2 binding ability and potentially improved bone regeneration performance.

## 5 Application of Heparin for Orthopedic Biomaterials Design

### 5.1 Orthopedic Nanomaterials

Bone tissue engineering is an excellent method to treat bone defects, and its efficiency has been improved with the development of orthopedic nanomaterials. Electrospun nanofibrous scaffolds and hydrogel scaffolds simulate natural ECM of bone tissue, enhance vitality and function of cells, promote formation of osteoblasts, and stimulate process of vascularization (Qasim et al., 2019). Nano/nanocomposites for bone regeneration form a class of easily absorbable orthopedic fillers by combining nanoscale bioactivity with biopolymeric and degradable matrix structures. Its design was based on a variety of materials, including metal (including nano zirconia, silver nanoparticles, and nano titanium dioxide), ceramics (nano hydroxyapatite and nano silicon dioxide), natural polymers (including chitosan, collagen, cellulose, silk fibroin, and alginate), high-molecular polymers [polycaprolactone (PCL), poly(lactic-co-glycolic) acid (PLGA), polyethylene glycol (PEG), poly(lactic acid) (PLA)] (Bharadwaz and Jayasuriya, 2020), and carbon-based nanomaterials such as graphene and its derivatives, carbon nanotubes and carbon dots (Peng Z et al., 2020). The modification of nanomaterials (such as large surface area, enhanced mechanical strength and stability, improved cell and drug adhesion, and delivery) is more favorable to the development of unique therapeutic strategies for specific bone defects (Peng Z et al., 2020). In addition, nanomaterial particles are essential in cell labeling and drug and gene delivery. Indeed, it can highlight special potential for mesenchymal stem cells, local release, and timing control in bone tissue (directly affecting osteoblasts and osteoclasts), and as a carrier of genetic material to overcome the limitations of traditional methods (Tautzenberger et al., 2012). Magnetic composite scaffolds formed by magnetic nanoparticles (MNP)–activated multiple signaling pathways (including MAPK, integrin, BMP, and NF-κB), resulting in a 2- to 3-fold increase in osteogenic differentiation, angiogenesis, and bone regeneration (Xia et al., 2018).

### 5.2 Nanomaterials Binding With Heparin

Due to the inherent nature of heparin to reversibly bind positively charged proteins, it is able to mediate continuous delivery of GFs (in particular VEGF, PDGF-BB, FGF, and BMP-2/4/6/7) while maintaining protein bioactivity (Biran and Pond, 2017; Hettiaratchi et al., 2017). In combination with biomaterials, heparin promotes angiogenesis and bone formation in bone tissue engineering, a well-researched field that aims to improve delivery of GFs for bone regeneration (Hettiaratchi et al., 2017; Rindone et al., 2019).

The use of heparin as a delivery mode may contribute to solving the limitations of nanomaterials used in bone engineering such as low growth factor retention or reduced growth factor activity. Nanomaterials with coated or electrodeposited heparin showed stable GF release without damaging the nanostructure and internal folding skeleton. Furthermore, these scaffolds were better than conventional scaffolds in bridging tissue gaps and reproducing the characteristics of the extracellular environment (Lee et al., 2017). In addition, magnetostrictive nanoparticles and biomimetic heparin coatings can change their shape according to the properties of the external magnetic field to better adapt to changes in the environment of bone regeneration (Guillot-Ferriols, et al., 2020). Nanosilver, antibiotics, or specific genes can cooperate with heparin composite nanomaterials and have excellent antibacterial and anti-inflammatory effects, or can enhance osteogenic signaling activation (Li et al., 2021; Coelho et al., 2015; Xie C. M et al., 2014). Different bonding modes between heparin and nanomaterials (e.g., nanocrystalline hydroxyapatite) produced different heparin chain conformations, which will affect the stability of heparin on nanomaterials and subsequent release of GFs (Konar et al., 2019). Therefore, the assembly of heparin and nanomaterials can control the release of growth factors by changing the way they interact with each other.

### 5.3 Application of Different Biomaterials in Bone Regeneration

It is critical to develop effective carrier systems for therapeutic compounds for bone tissue engineering applications (Zhang et al., 2018). The ideal carrier should be biocompatible, efficient in drug-release, and preserve the compound bioavailability. The combination of heparin with the carrier should provide a stable, persistent, and targeted-controlled release of the drug (Liang and Kiick, 2014). Currently, various nanomaterials are being used for bone repair, including metallic, inorganic, organic, and natural polymers (Sakiyama-Elbert 2014) with heparin incorporated through surface coating, covalent binding, and biomimetic delivery (Hettiaratchi et al., 2014; Johnson et al., 2011; Kim et al., 2015a).

#### 5.3.1 Heparin for Metallic Nanomaterial Surface Coating

The binding of heparin to titanium (Ti) surfaces has been extensively studied. The heparin/BMP-2/Ti complex enhanced osteogenic activation of MG-63 cells, and the heparin/BMP-2/Ti complex increased ALP activity and calcium deposition compared with pristine Ti surfaces (Lee S. Y et al., 2013). Similarly, Kim et al. demonstrated that Ti/Hep/BMP-2 increased OCN and OPN levels in differentiated osteoblasts. Moreover, BMD and bone to implant contact ratio were significantly greater with Ti/Hep/BMP-2 substrates compared to Ti *in vivo* (Kim S. E et al., 2014). The aminated titanium exhibited enhanced heparin binding and augmented release kinetics of BMP2 over a 28-day period compared with Ti only resulting in superior osteoinductivity (Kim E. C et al., 2014). In addition, downregulation of TNF-α and IL-6 mRNA was also detected in cells grown on Ti/Hep/BMP-2 compared with those on heparin-grafted Ti. This suggests that Ti/Hep/BMP-2 should also have beneficial anti-inflammatory effects on osteogenesis (Kim et al., 2011). In another study, the addition of gentamicin sulfate to Ti/Hep/BMP-2 significantly inhibited bacterial infection and enhanced ALP activity and calcium deposition of osteoblasts. Therefore, dual drug-eluting (antibiotic and bone-inducing protein) Ti substrates are beneficial for improving the prognosis of orthopedic implants (Lee D. W et al., 2012).

Silver- and GF-doped hydroxyapatite-coated Ti implants showed enhanced osteoinduction capacity and antibacterial properties. BMP-2, chitosan, and heparin were adsorbed on the coat by electrostatic attraction to ensure continuous release of BMP-2 and silver ions, and it demonstrated excellent antibacterial capacity against *Staphylococcus epidermis* and *Escherichia coli*, as well as efficient osteoinduction (Xie C. M et al., 2014). Therefore, GFs binding to antimicrobial agents on the surface of metal implants is a simple and effective method to promote bone formation.

#### 5.3.2 Heparin for Inorganic Nanomaterial

Bioinorganic non-metallic materials include bioglass, bioceramic, bioactive cement, and bioceramic cement. They are characterized by excellent chemical stability, histocompatibility, high compressive strength (which plays a role in bone healing through surface modification), covalent action, and biomimetic delivery of heparin (Fahimipour et al., 2019).

##### 5.3.2.1 Surface Coating

Mesoporous bioactive glass (MBG) is an excellent bioceramic for bone transplantation. To recapitulate an ECM-like surface, scaffolds using decellularized matrix from porcine small intestinal submucosal (SIS) provides a plane that can enhance self-renewal, proliferation, and differentiation of stem cells. By heparinizing MBG/SIS scaffolds and conjugating P28, a BMP-2–related peptide (MGB/SIS-Hep-P28), the release of P28 was extended (40 days), resulting in increased cell proliferation, viability, ALP activation, and enhanced expression of osteogenesis-related genes (including Runx2, OCN, OPN, and ALP) *in vitro*. Furthermore, MGB/SIS-Hep-P28 scaffolds significantly increased bone formation of rat calvarial critical-size skull defects compared with controls *in vivo* (Zhou et al., 2020).

Similarly, loading heparin/collagen multilayer films on bidirectional calcium phosphate (BCP) allows for the programmed release of BMP-2. The heparin/collagen multilayer on BCP reduced the initial release of BMP-2 by >50% and loaded more BMP-2 during the differentiation phase of osteogenic cells. This suggests that heparin loading on the surface of orthopedic biomaterials is more favorable for the stability and durability of bone formation (Han et al., 2021).

One promising strategy for promoting angiogenesis in bone tissue is local and continuous release of angiogenic factors. Varying the amounts of heparin within the heparin-modified biocement (Bio D)/collagen type I (Col-I) complexes resulted in diverse effects on the release of VEGF. Increasing heparin inhibited the initial burst of release in a concentration-dependent manner and promoted VEGF activity and improved BioD/coll biological properties. In addition, the heparin-modified BioD/coll composite showed a finer microstructure, with smaller heparin particles and higher specific surface area, making it more favorable as a bone graft substitute for bone healing (Lode et al., 2008).

##### 5.3.2.2 Covalent Bindings

The performance of heparin can be affected by how it is coupled to a substrate. Goonasekera et al., assessed the impact of how heparin attached to the surface of hydroxyapatite particles affected the rate of BMP-2 release. When heparin was covalently attached to the hydroxyapatite surface *via* a (3-aminopropyl)triethoxysilane (APTES) layer, the release rate of BMP-2 was 31% after 7 days. This is compared with release rates of 16 and 5% when heparin was adsorbed to APTES-modified particles or pure hydroxyapatite, respectively. Consequently, the release curve and total amount of BMP-2 can be controlled by changing the attachment mode between heparin and a biomaterial (Goonasekera et al., 2015).

Another method of enhancing the regeneration of bone *via* heparin-mediated mechanisms is to conjugate heparin with 1-amino-1,1-diphosphonate methane (aminoBP) to enhance its affinity to hydroxyapatite. Increasing the number of conjugated aminoBPs resulted in a > 2-fold increase in the affinity of heparin for hydroxyapatite *in vitro*. Moreover, aminoBP-heparin conjugants were investigated, and they could enhance bone mineral affinity of bFGF and BMP-2. The authors found that conjugated heparin increased bone mineral affinity of these GFs in an aminoBP-dependent manner indicating that combining aminoBP with heparin improves the affinity of osteogenic GFs for hydroxyapatite (Gittens et al., 2004).

##### 5.3.2.3 Biomimetic Delivery

Biomimetic delivery systems are important for bone regeneration. Heparin was covalently connected to cross-linked type I collagen–coated tricalcium phosphate/hydroxyapatite and loaded with BMP-2 to form a local continuous delivery system of BMP-2 to improve bone regeneration for the treatment of large bone defects (Hannink et al., 2013). Delivery of BMP-7 from a bioactive glass/heparin/gelatin nanocomposite scaffold in rat calvarial critical-size skull defects induced fully mature new bone at the site of injury at 12 weeks suggesting synergistic effects of cells, scaffolds, and growth factors in bone regeneration (Kargozar et al., 2017). Jo et al., hypothesized sequential delivery of BMP-2 and BMP-7 would enhance bone regeneration more effectively than BMP-2 alone. The sequential delivery of BMP-2/7 with heparinized collagen membranes showed significant induced new bone formation in rat calvarial defects compared with single delivery of BMP-2 or BMP-7 (Jo et al., 2015). Another composite scaffold is CaCO_3_ microspheres which possess osteoinductivity, rough surfaces, and specific binding sites for BMP-2. When encapsulated in heparin fibrin hydrogel, osteogenic differentiation of BMSCs was augmented (Gong et al., 2019). In summary, different delivery systems can affect bone regeneration by regulating GF delivery (Wang et al., 2018).

Alginate sulfate, a synthetic heparin structure mimic, has similar bioactivity to heparin and is used in three-dimensional (3D)-printed scaffolds. This 3D micropatterning method can be applied to different heparin-binding GFs (including FGF-2, VEGF, TGF-β, and BMP) while retaining the natural degradation and cellular compatibility of hydrogels. Stem cells loaded in micropattern hydrogels exhibited spatially localized growth and differentiation responses corresponding to various GFs patterns, suggesting the adaptability of using 3D micropatterning to control stem cell behavior in bone tissue engineering (Jeon et al., 2018).

#### 5.3.3 Synthetic Polymer

##### 5.3.3.1 Surface Coating

Biosynthetic organic materials are widely used in the field of orthopedics with heparin-coated organic materials demonstrating benefits such as sustained protein release, favorable biocompatibility, high growth factor loading rate, and precise control (Hamideh et al., 2020; Nakamura et al., 2020).

Improvements in the development of coacervate particles have included the electrostatic complexation between poly(ethylene arginyl aspartate diglyceride) (PEAD) polycations and anionic heparin (termed “coacersomes”) to avoid aggregation and regulate the release of BMP-2. The coacersomes demonstrated biocompatibility with human dermal fibroblasts, a high loading efficiency (> 96%) for encapsulated BMP-2, sustained release of up to 28 days, and increased osteogenic differentiation of hMSCs (Jo et al., 2019).

##### 5.3.3.2 Covalent Binding

Although PCL fiber scaffolds are inert, the surface incorporation of heparin and BMP-2 (PCL/Hep/BMP-2 scaffold) can promote rapid and stable integration of adjacent bone tissue. In these scaffolds, the concentration of BMP-2 increased systematically with the incorporation of heparin, and its efficacy was preserved through covalent binding, facilitating MSC proliferation and increasing ALP activity, deposition of bone sialoprotein, OPN, and calcium minerals deposition (Gadalla and Goldstein, 2020).

Insufficient vascularization is an important limitation in engineering porous scaffolds in tissue engineering. Heparin cross-linked with N-hydroxysuccinimide and N-(3-di-methylaminopropyl)-N'-ethylcarbodiimide resulted in demineralized bone matrices (DBM) being able to bind more VEGF, and this achieved localized and sustained delivery compared with non-cross-linked scaffolds. VEGF was biologically active when bound to heparinized DBMs demonstrated by increased proliferation of endothelial cells and improved angiogenesis thus augmenting vascularization and bone regeneration (Chen et al., 2010).

##### 5.3.3.3 Biomimetic Delivery

Currently, the biomimetic delivery systems of heparin/organic polymer materials have been studied extensively using hydrogels (Sievers et al., 2019), scaffolds (Jeon et al., 2007), fibers (Lü et al., 2018), collagen (Vantucci et al., 2021), and nanospheres (Reguera-Nuñez et al., 2014). Multiple organic materials loaded with GFs at the bone defect have achieved long-term sustained release in the target area and induced bone formation. In Li et al., PEAD and heparin were complexed to form a novel platform to release and deliver BMP-2, which promoted differentiation of myogenic stem cells into an osteogenic lineage (Li et al., 2013). Thin film materials assembled by using the layer-by-layer (LBL) method are highly adaptable and versatile in terms of the type of substrates and polyelectrolytes (such as GFs) that can be used for bone tissue engineering. Polyelectrolytes provide the initial charge necessary for multilayer construction with counter polyelectrolytes such as heparin being able to enhance the activity of GFs by protecting their ligands. This produced the highest loading efficiency for the tested GFs (Damanik et al., 2019) suggesting the LBL assembly method can improve the delivery system of biological molecules for bone tissue engineering applications.

The addition of a heparin-based hydrogel to porous cylinder poly (L-lactide-co-ε-caprolactone) (PLCL) scaffolds effectively accelerated the maturation and differentiation of fibrochondrocytes, and was used for local release of fibrochondrocytes and BMP-2 to the fibrochondral region (Lee et al., 2011). Photocrosslinkable biomaterials such as alginate saline gel (Hep-ALG) have been harnessed to develop a controlled, prolonged release of BMP-2 with the addition of heparin to the hydrogel (Jeon et al., 2011). Another hydrogel system containing hyaluronic acid which included heparin also supported osteoblast growth (> 8 weeks) and sustained BMP-2 release (> 35 days) in rats (Rath et al., 2011). Compared with hydrogels without heparin, it inhibited the initial burst of BMP-2 and maintained BMP-2 activity for up to 28 days (Bhakta et al., 2012). Other scaffolds use sulfonated hydrogels to mimic heparin [such as poly(vinylsulfonic acid) (PVSA) or poly-4-styrenesulfonic acid (PSS)] which was found to effectively isolate and stabilize BMP-2 to enhance bone induction activity (Kim S et al., 2018).

When hMSCs were cultured on top of heparin-conjugated PLGA (Hep-PLGA) scaffolds loaded with BMP-7/TGF-β3 nanocomplexes, they showed cartilage formation macroscopically and histologically (Crecente-Campo et al., 2017). BMP-2 released by Hep-PLGA stimulated ALP activity for approximately 14 days, increased bone formation area 9-fold, and induced calcium content 4-fold compared with unmodified heparin PLGA scaffolds (Jeon et al., 2007). PCL/PLGA scaffolds were conjugated with heparin and dopamine to form a scaffold and coated with BMP-2*. In vitro* studies with osteoblast-like MG-63 cells cultured on the scaffolds demonstrated significantly enhanced ALP activity, calcium deposition and bone formation on BMP-2/Hep-DOPA/PCL/PLGA scaffolds compare to those without dopamine. It is suggested that dopamine may enhance the osteogenic effects in conjunction with heparin (Kim T. H et al., 2014). Hep/PCL/gelatin scaffolds also provide a controlled release of PDGF-BB and prolong the bioactivity of the molecule, therefore facilitating angiogenesis (Lee J et al., 2012). Another delivery system involves using polyelectrolyte multilayer films to release FGF-2 locally, precisely, and continuously. The presence of counter polyanion, HS in the multilayer structure enhanced FGF-2 osteogenic activation (Macdonald et al., 2010).

The GF delivery carrier formed by the heparinization modification of polylactic acid (PLA) is termed fiber particles (Hep-FP). Hep-FPs showed stable BMP-2 binding and sustained release, augmented ALP activity, hMSC mineralization, and higher BMD formation in the defect area (Shin et al., 2015). Combination of heparin-mediated bFGF into PCL/gelatin fiber mesh also had the ability to direct bone regeneration (Lee J. H et al., 2015). VEGF and heparin were fixed in PLGA nanofibers, which induced vascular formation of MSCs and were more favorable for bone regeneration (Lü et al., 2018).

Nano-microspheres, nanosponges, and nanocomplexes can effectively bind several heparin-binding GFs (including BMP-2, PDGF-BB, VEGF, FGF-2). Most of them are prepared with heparin and PLGA. BMP-2 or BMP-7 incorporated into microspheres remain biologically active and effectively initiate functional cellular responses (Hettiaratchi et al., 2014; Reguera-Nuñez et al., 2014), which significantly induced ALP activity, calcium deposition, OCN and OPN expression (Kim et al., 2015b; Liu et al., 2016). The GF delivered by PDGF-BB nanoparticles accelerated the generation of blood vessels, meeting the key requirements of bone regeneration (d'Angelo et al., 2010; La and Yang, 2015). Meanwhile, the release of FGF-2 was prolonged by heparin nanosponges and stimulated the growth and differentiation of hBMSCs (Choi W. I et al., 2017; Shin et al., 2014).

#### 5.3.4 Natural Polymers

##### 5.3.4.1 Surface Coating

Natural polymer materials include chitosan, collagen, silk protein and cellulose. The chitosan/agarose/gelatin (CAG) scaffold was modified with heparin and loaded with stromal cell derived factor 1 (SDF-1) and BMP-2. SDF-1 and BMP-2 released retained bioactivity and induced the sustained recruitment and differentiation of MSCs (Wang et al., 2018). Heparin-modified collagen scaffolds can promote the transmission of BMP-2 to bone defects and reduce heterotopic ossification in critically sized femoral defects in a rat model (Hettiaratchi et al., 2020). BMP-2 released by heparin-conjugated fibrin (HCF) induced higher BMP-2 retention, ALP activity and BMD compared with collagen sponges (Yang et al., 2012).

##### 5.3.4.2 Covalent Binding

The heparin analogue, dextran sulfate (DS) is covalently linked to chitosan to form nanoparticles. Heparin-binding proteins (SDF-1α VEGF, FGF-2, BMP-2.) were added, and SDF-1α and VEGF demonstrated full activity and sustained heat stability. The other GFs exhibited good osteogenic effects due to their heparin-binding sites (Zaman et al., 2016). On the other hand, neither heparin nor BMP-2 alone promoted bone growth within collagen scaffolds. BMP-2 collagen scaffolds complexed with heparin stimulated new bone regeneration with similar mechanical properties to intact bone (Johnson et al., 2011).

##### 5.3.4.3 Biomimetic Delivery

Heparinized chitosan has a protective effect on BMP bioactivity and can resist the physiological stressors related to fracture healing. It enhanced osteogenesis by inhibiting Noggin activity and attracted BMSCs (Kim et al., 2020). Moreover, it exhibited synergetic effects between BMP-2 and VEGF, which effectively augmented bone formation stimulating osteogenesis and angiogenesis (Zhang S et al., 2019). Heparinized chitosan can stably adsorb FGF-2 to the surface and release it into the bone defect (Zomer Volpato et al., 2012). In addition, stabilizing FGF-2 in the interior of chitosan with a heparin-based nanoparticle complex can preserve its activity and stimulate BMSCs (Place et al., 2016). The gelatin/chitosan frozen gel surrounding the heparin/gelatin frozen gel exhibited different drug release kinetics. Early release of VEGF and continuous delivery of BMP-4 can induce successful osteogenic differentiation *in vitro*. The dual release leads to the enhanced effect of bone regeneration (Lee et al., 2020). In addition, whitlockite enhanced VEGF secretion of human adipose stem cells inoculated with heparin/gelatin. The sustained release of VEGF was observed, which promoted angiogenesis to enhance bone formation *in vivo* and significantly increased bone regeneration (Kim et al., 2019).

The BMP-2 delivery using heparin-conjugated collagen sponges (HCS) had a low initial burst and then a sustained BMP-2 release. Over time, HCS-BMP scaffolds guided more efficient bone regeneration within the defect, as well as ossification outside the defect (Kim et al., 2016). When combined with a matrix derived SDF-1α, the concentration of BMP-2 could be reduced since SDF-1α could enhance the osteoinduction ability of BMP-2 (Zwingenberger et al., 2016). Compared with the conventional collagen scaffold used in clinic, the mixed scaffold composed of heparin, BMP-2, nanofiber, and fibronectin was more effective in bridging the gap (Lee S. S et al., 2013). Heparin-conjugated collagen also acted as a carrier to deliver PDGF-BB, which stimulated angiogenesis. VEGF-preloaded heparin collagen scaffolds also promoted the formation and stabilization of prevascular structures (Quade et al., 2017). Therefore, heparinized collagen coupled with GF can significantly improve angiogenesis and bone regeneration.

HCF systems provided long-term release of BMP thus enhancing bone regeneration, stimulating ALP activity, increasing OCN level and the ratio of calcium to phosphorus in the regenerated bone, as well as producing more mature and highly mineralized bone than bare fiber gel (Chung et al., 2007). In addition, the controlled delivery of recombinant BMP-2 stimulated by HCF inhibited the formation of adipose tissue in the defect area and enhanced mineralization, thus greatly alleviating the side effects of adipose bone marrow formation caused by high concentration of recombinant BMP-2 (Lee J. S et al., 2015).

#### 5.3.5 Composite Materials

Combining multiple composite materials has been found to produce better results than using individual materials. Combining inorganic and natural polymer materials provides enhanced mechanical properties as well as thermal stability. Heparin coupled to a strontium-substituted hydroxyapatite/silk scaffold was found to increase proliferation and adhesion of BMSCs, upregulate expression of osteogenic genes including OCN and OPN, and increase BMD, thereby enhancing new bone regeneration (Yan et al., 2018).

Hydroxyapatite was coated with collagen to mimic the composition of bone and enhanced cell attachment, proliferation, nutrient transport, and infiltration of new bone tissue. Combining heparin and a mineralized collagen matrix is important to maintain the activity and sustained release of loaded BMP-2 and VEGF. It can stimulate angiogenesis and bone regeneration through GF binding and favorable release properties (Knaack et al., 2014).

Hybrid materials comprising inorganic and metallic materials promoted increased bone volume, bone volume/tissue volume, and better bone remodeling characteristics than the individual materials (Yang et al., 2015). Incorporation of metal particles such as silver into hydroxyapatite coating can increase the antibacterial properties in addition to osteogenesis. Furthermore, chitosan can not only stabilize chelation but reduce the toxicity of silver ions (Xie C. M et al., 2014).

**FIGURE 4:**
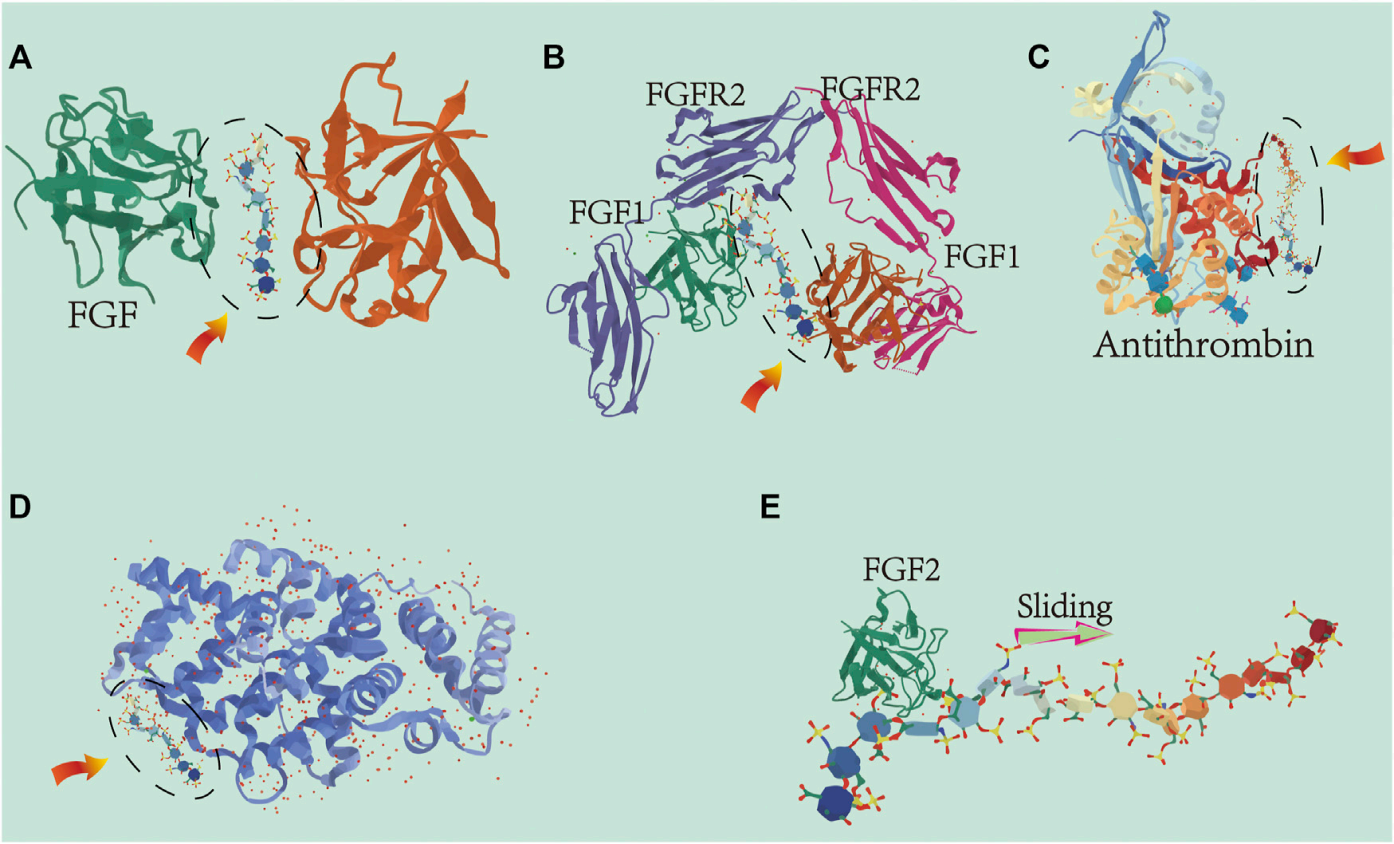
Cartoon representation of the different ways heparin interacts with growth factors: The heparin oligosaccharide unit (highlighted by the dashed oval) is located relative to the heparin-binding site within the proteins structure of the growth factors. **(A)** Heparin-induced dimerization of heparin-binding protein. Numerous members of the FGF family interact with heparin to form dimers. Protein–heparin interactions drive the dimerization in the absence of protein interactions at the dimer interface. **(B)** Heparin acts as a scaffold for protein–protein interactions for efficient binding and regulation between proteins. The eutectic structures of FGF-1, FGFR2, and heparin-derived decosaccharides are shown here. **(C)** Heparin acts as an allosteric regulator. The unique pentasaccharide within heparin binds to antithrombin and induces allosteric changes mitigating the actions of serine protein C inhibitors which inhibit antithrombin activity. **(D)** Interaction between heparin and growth factors (such as BMP and VEGF) is dependent on salt concentration. Binding affinity increases with increasing oligosaccharide length. **(E)** Ability of heparin to bind proteins to specific locations in tissues. The length and flexibility of the heparin chain allows bound growth factors (e.g., FGF-2) to move unidirectionally along the chain and ensure localization of the bound growth factors.

The incorporation of gold nanoparticles into poly-l-lysine heparin membranes showed enhanced mechanical properties (Qi et al., 2017). The macroporous scaffold composed of chitosan, hydroxyapatite, heparin and polyvinyl alcohol resulted in a more uniform matrix structure, and has the mechanical properties to promote bone regeneration (Sultankulov et al., 2019). In summary, the combinations of the appropriate materials have synergistic GF induction properties and provide a better microenvironment for bone healing. Therefore, multifunctional membranes as bone induction coatings for biomaterials have far-reaching significance. The effects of heparin on GFs are summarized in Figure 4.

## 6 Conclusion

In this review, we have summarized the recent advances of how heparin is utilized in bone tissue engineering, including the regulation of osteogenic GFs and bone regeneration materials. We highlighted the use of heparin as a multifunctional material modifier to promote angiogenesis and osteogenesis. Heparin provides a range of features (including the regulation of GFs BMP, FGF-2, PDGF-BB, and TGF-β and their applications in biomaterials) that are important for effective GF release, angiogenesis, and bone formation. In addition to heparin, there are numerous other substances or molecules that can bind GFs for bone regeneration. For example, bisphosphonate-loaded nanomaterials achieved controlled and sustained delivery of GFs (Fanord et al., 2011). In addition, molecules such as peptides (e.g., AspSerSer6) or plasmid DNA can effectively deliver transcription factors and GFs to induce osteoblasts and thereby enhancing bone formation (Zhang et al., 2012). Moreover, interleukin-1 receptor antagonist-binding nanomaterials reduced inflammation and reversed bone turnover (Fernandes et al., 2008). However, the use of heparin is a more promising area than the aforementioned methods because of its improved safety, degradability, surface modification capacity, and easier space application.

The specific binding site within heparin that mediates interaction with a variety of osteogenic GFs improves neovascularization and bone tissue regeneration. Compared with other anticoagulant drugs, heparin-modified bone regenerative materials have higher osteogenic activity, which is favorable for the delivery and retention of therapeutic GFs. This is closely related to the fact that heparin is a negatively charged natural polysaccharide with a specific binding site that binds GFs, which prevents their degradation and also has anti-inflammatory and angiogenic effects. Heparin-modified biomaterials (particles, fibers, films, and 3D scaffolds) can be used as GF vehicles to transport drugs/GFs expressing angiogenic and osteogenic capabilities, augmenting kinetic release and decreasing side effects due to the inherent biological activity of heparin. By effectively stimulating bone formation through the release of therapeutic drugs/GFs, the delivery of heparin modified materials may serve as a more widespread platform for bone and cartilage regeneration. We also discussed the control of heparin on the initial burst of GFs and the importance on sustaining the subsequent release. More focus should center on the relative merits of these GFs and their application forms (as particles or scaffolds), dosing for different cell lines or animal models as well as optimizing the specific combination of GFs and materials to achieve synergistic effects while managing release kinetics. Future research should concentrate on the development of heparin-modified materials with inflammatory components that simultaneously promote the coupling of angiogenesis and osteogenesis.

Although heparin-modified material carriers have many advantages, limitations need to be addressed. For example, the drug conjugated to heparin does not maintain the same biological characteristics *in vivo* after delivery, the effective charge may be unstable *in vivo,* and chemical conjugation may also affect the non-covalent interaction of the carrier in some heparin-modified materials. Therefore, multilayer slow-release systems can be designed to make the release of drugs or GFs more stable. Furthermore, the addition of a constant magnetic field to the outside of the material may promote directional force to ensure steadier drug release. The bioactivity of GFs plays a critical role in bone regeneration, and the application of heparin in biomaterials is the key to maintaining GF activity. Heparin can effectively protect the biological activity of GFs at the site of bone injury and ensure a controlled release during the treatment for maximum effects. Therefore, heparin modification is a promising approach in bone tissue engineering biomaterial development. In addition, directions for future research should include but not limited to investigating the optimal components for stimulating bone formation: 1) the particular effects of heparin-specific therapeutic GF dissolution and its relevant concentrations on angiogenesis; 2) develop standard *in vitro* models to characterize multifunctional biomaterial systems that reflect the participation of the immune system *in vivo*; 3) heparin-modified composite scaffold with multiple GFs and multiple materials; and 4) develop appropriate animal models to study heparin-induced new bone tissue and blood vessels. It is likely that in the future, advances in the understanding of osteogenic regulation of heparin and the advances in composite material research will further enhance the appeal of using heparin clinically for the treatment of bone tissue defects.
